# Detecting polystyrene nanoplastics using filter paper-based surface-enhanced Raman spectroscopy†

**DOI:** 10.1039/d2ra03395j

**Published:** 2022-07-15

**Authors:** Shinji Kihara, Andrew Chan, Eugene In, Nargiss Taleb, Cherie Tollemache, Samuel Yick, Duncan J. McGillivray

**Affiliations:** School of Chemical Sciences, The University of Auckland Auckland 1010 New Zealand d.mcgillivray@auckland.ac.nz; The MacDiarmid Institute for Advanced Materials and Nanotechnology Wellington 6140 New Zealand

## Abstract

This work presents a novel filter paper-based method using surface-enhanced Raman spectroscopy (SERS), for detecting polystyrene nanoplastics (PSNPs). The SERS system used a simple mixture of spherical Au nanoparticles (AuNPs) and 20 nm nanoplastics deposited onto a filter paper which offered a detection limit of 10 μg mL^−1^ with a sample volume of 50 μL, and in a rare case 5.0 μg mL^−1^ (with four aliquits of 50 μL).

## Abstract 2

This work presents a novel filter paper-based method using surface-enhanced Raman spectroscopy (SERS), for detecting polystyrene nanoplastics (PSNPs).

## Graphical abstract

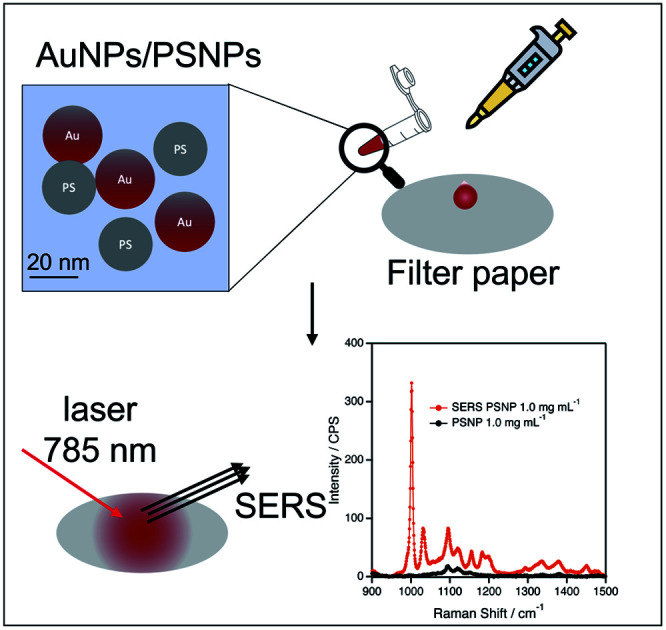

The global threat of accumulated plastic pollution at the nanoscale has recently raised concerns from both the scientific^1,2^ and regulatory^3,4^ communities. The potential impacts include toxicity on human, aquatic and terrestrial ecosystems.^1,5–7^ Although their larger analogues, microplastics, have gained more extensive coverage in the literature,^1^ the nature of problems presented by nanoplastics (*e.g.*, biological and chemical interactions in the environment) may inherently differ from those of microplastics.^2^ Nanoscale plastic particulates (*e.g.* ∼20 nm) like polystyrene (PS) can bioaccumulate at the cellular level, interact with chromosomes and DNA, and can prompt a ‘trojan-horse’ toxicity effect with co-contaminants.^8^

Despite the recent expanse of nanoplastic toxicology research, the field remains deprived of standardised detection methods which makes building robust risk assessment frameworks challenging.^9^ Currently, the most common analytical methods employed for nanoplastic detection are scanning electron microscopy (SEM) and transmission electron microscopy (TEM), Fourier-transform infrared spectroscopy (FTIR), mass spectrometry (MS) and Raman spectroscopy.^10^ Unfortunately, each technique faces resolution limits at the nanoscale and as such more efficient means of observing nanoplastics must be developed.

Surface-enhanced Raman spectroscopy (SERS) on a confocal microscope offers a high spatial resolution with minimal sample preparation.^11^ The SERS technique, in general, uses a nanostructured plasmonic material, typically consisting of gold and/or silver, which can significantly enhance the Raman scattering signals from resonant interactions with analytes. Consequently, SERS compensates for the inherently low Raman scattering cross sections and concentrations of nanoplastics.

Reported systems in the literature typically use a SERS active colloidal system for *in situ* measurements^12,13^ or carefully designed nanostructured surfaces.^14,15^ While sample preparation for colloidal systems is comparably simpler, the limit of detection (LOD) is considered to be relatively high (∼40 μg mL^−1^). In contrast, nanostructured surfaces have demonstrated improved effectiveness (LOD = 10 μg mL^−1^), but the fabrication of such substrates are difficult.

This work presents a filter paper-based SERS substrate (Fig. 1(a)) which combines the advantages of colloidal and nanostructured solid systems, boasting a simple production method with good sensitivity. Particular advantages attributed to filter paper-based SERS are the ability to: create greater hot-spot sites, concentrate analyte, and ability to separate non-target molecules (if required).^16^ Filter paper-based SERS substrates have recently shown strong capability in detecting trace amount of small molecules^16^ while a cellulose-based substrate has been reported as a ESI† for SERS active NPs.^17^ To our knowledge, this study is the first account of incorporating AuNPs on filter paper substrates that demonstrates the SERS capability with polystyrene nanoplastics (PSNPs).

**Fig. 1:**
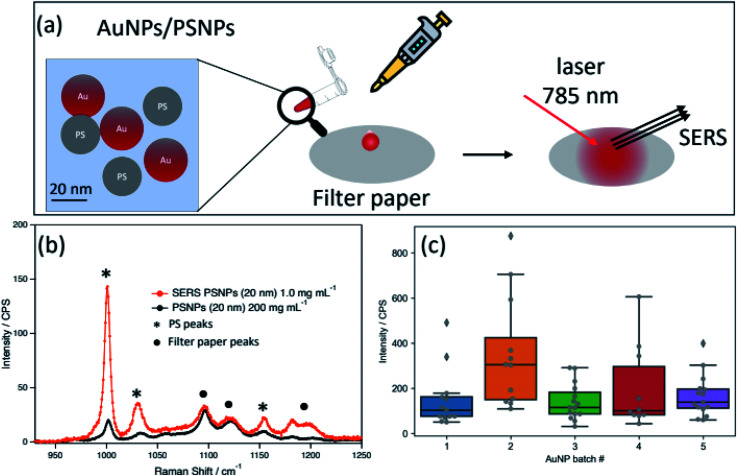
(a) Schematic illustration for the filter paper system developed in this work, (b) representative surface-enhanced Raman spectra for PS(+)20 (1.0 mg mL^−1^) with AuNP2 and unenhanced for PS(+)20 (200 mg mL^−1^), and (c) box plots of surface-enhanced Raman intensities at 1004 cm^−1^ from each AuNP batch.

Spherical AuNPs were synthesised *via* a Reversed Turkevich method.^18^ As SERS performance is correlated to nanoparticle monodispersity and shape uniformity, synthetic conditions (*e.g.*, chloroauric acid concentration) were varied to find optimum values (Table S1†). All AuNP batches showed an intense absorption peak at approximately 518 nm (Fig. S1†), with AuNP2 having the strongest absorption. Size and shape qualities were assessed using dynamic light scattering (DLS, Table S1†) and transmission electron microscopy (TEM, Fig. S2†). Of all batches, AuNP1 and AuNP2 showed the best polydispersity index (PDI) of 0.45 and 0.45, respectively, while shape analysis on AuNPs imaged by TEM (Fig. S2, S3, and Table S1†) showed more uniform diameter distribution for AuNP2 than AuNP1 (4.2 *vs.* 6.7 nm). Nevertheless, all the synthesised AuNP batches were highly spherical in shape with little variation across each batch (aspect ratios all close to 1.0).

Prior to SERS experiments, control Raman spectra of bulk polystyrene and filter paper were collected to identify their respective vibrational peaks when detecting PSNPs. Notable Raman signals from PS (Fig. S4†) include the aromatic ring C–C stretching (1004 cm^−1^), C–H in-plane deformation (1030 cm^−1^), and aromatic ring C=C deformation (620 cm^−1^).^19^ From filter paper (Fig. S2†), the signals are attributed to cellulose; glycosidic linkage stretching modes (1095 and 1120 cm^−1^)^20^ and C–C stretching mode (995 cm^−1^).^21^ Considering the relative strength of the observed peaks from PS and lack of complete signal overlap (Fig. S4 and S5†), we selected the Raman peak at 1004 cm^−1^ as the reference peak for our discussions.

Selective enhancement of analyte signals over the filter paper matrix or the capping agent on the AuNP surface is key to the development of successful SERS substrates. The initial set of SERS experiments (Fig. S5†) using AuNP2 and positively charged PSNPs (PS(+)20) at 1.0 mg mL^−1^ showed neither a significant change to the characteristic peaks observed in the control samples nor an enhancement of trisodium citrate (capping agent) and filter paper (Fig. S2†). Additionally, a proportional enhancement was observed for all PS peaks. These observations collectively suggest: 1. AuNPs and PSNPs do not chemically interact with the filter paper matrix; and 2. covalent bonding is not the enhancement mechanism for this system (preferential enhancement of specific vibrational peak is expected otherwise); physisorption and hotspot field creation by AuNPs are likely pathways.

To assess SERS performance and calculate the enhancement factor (EF) of each AuNP batch, Raman spectra (Fig. 1(b)) for PS(+)20 (1.0 mg mL^−1^) mixed with AuNPs (Au concentration fixed at 0.05 mg mL^−1^) were collected at fixed instrument parameters (*e.g.*, laser power and confocal hole size, details found in the Method and Materials, ESI†). For the un-enhanced PSNP spectrum, the concentration was increased to 200 mg mL^−1^ to obtain sufficient signals at 1004 cm^−1^. The calculated EF (calculation method is shown in ESI†) for most AuNP batches was within the range of ∼1100–1750, except for AuNP2 with an improved EF of ∼3050 with an interquartile range of 1440 (∼50% variance) for AuNP2 (a list of EF and interquartile ranges for each AuNP is also available in the Table S3†). The origin of the higher EF of AuNP2 can be attributed to its more uniform diameter distribution of AuNP2 and the strongest plasmon band at 518 nm (Fig. S1†). Therefore, we, hereafter, only used AuNP2 for further SERS testing.

Next, the LOD for PS(+)20 was determined using AuNP2. The Raman spectra of AuNP2 at different PS(+)20 concentrations, varying from 500–1.0 μg mL^−1^, are presented in Fig. 2. The PS peak at 1004 cm^−1^, is apparent at concentrations as low as 10 μg mL^−1^. The broad peak observed at around 1000 cm^−1^ at 1.0 μg mL^−1^, indicated an overlap between the vibrational peaks from the PS and the pyranose backbone vibrations of filter paper. To distinguish each contribution, we compared this spectrum bare filter paper with Raman intensities normalised to the peak positioned at 1095 cm^−1^ (Fig. S6†). The difference between them was marginal, thus we conclude this system to have a LOD of 10 μg mL^−1^.

**Fig. 2:**
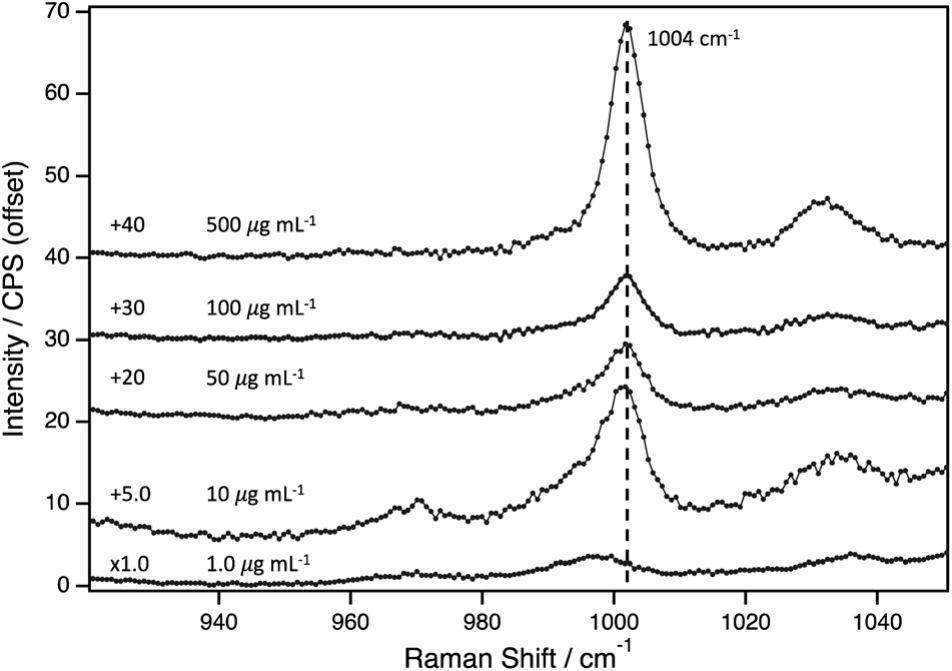
Surface-enhanced Raman spectra for PS(+)20 (concentration varied from 500–1.0 μg mL^−1^) with AuNP2 on filter paper. Each spectrum is an average of three independent sets of measurements from different spots.

Further, we tested the possibility of concentrating analyte by applying multiple aliquots with PS(+)20 (5.0 μg mL^−1^) and AuNP2. While most sampled spots showed no PS peaks, in a rare case, successful detection of PSNP was observed (Fig. S7 and S8†). We hypothesise that this enhancement is attributed to the formation of abnormal number of hot spots within the sampled region. Therefore, the one-off result is not representative and the mechanism underpinning such strong enhancement remains unclear (neither is this the scope of this work). However, future studies can reproducibly exploit this phenomenon to achieve even lower detection limit by redesigning (*e.g.*, optimising the AuNP/analyte ratio) and identifying the underlying enhancement mechanism.

These SERS experiments were repeated with different size (PS(+)200) and PSNPs bearing different surface charge (negative charge). With an increase to the analyte size, the LOD was significantly reduced to 1.0 mg mL^−1^ (Fig. S8†), possibly attributed to the reduced number of PS particles exposed to the SERS active AuNPs (*i.e.*, much of bulk mass of PS particles is not exposed to SERS hotspot). This observation is contrary to the reported trend in other SERS-based nanoplastic detection systems,^13,17^ where our detection limit was lower for larger nanoplastics. Furthermore, we observed that the enhancement effect was not apparent for the negatively charged PSNPs, irrespective of particle size (PS(−)20 and PS(−)200) (Fig. S9†). This quenching effect is likely attributed to the electrostatic repulsion between the AuNPs and PSNPs, preventing PSNPs to be in the SERS hotspot of AuNPs.

Last, the effect of NaCl concentration was tested on our SERS system (150 and 600 mM), to approximate ionic strengths of physiologically relevant biological fluid and seawater, respectively. At 100 μg mL^−1^, the Raman intensities at the 1004 cm^−1^ were marginally affected due to the presence of NaCl (Fig. S10†). However, the signal enhancement was completely quenched at 10 μg mL^−1^ of nanoplastic (Fig. 3) for both 150 and 600 mM, lowering the LOD to 100 μg mL^−1^.

**Fig. 3:**
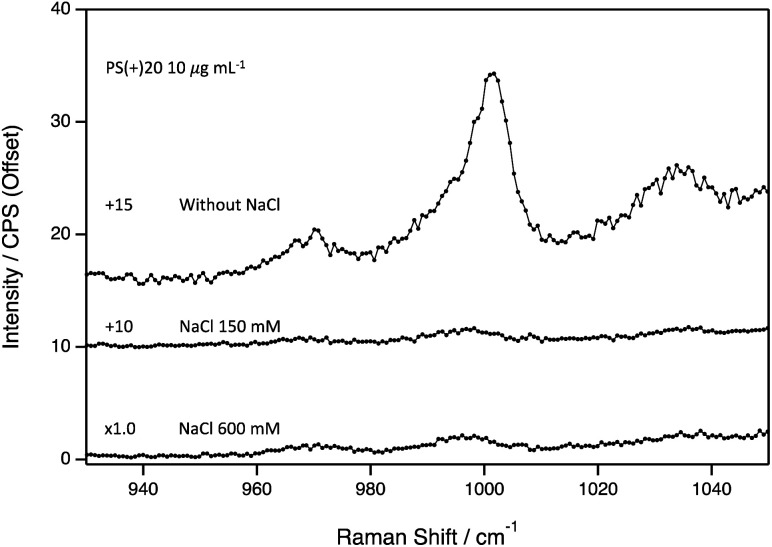
Surface-enhanced Raman spectra for PS(+)20 (10 μg mL^−1^) with or without NaCl (150 or 600 mM).

While our filter paper-based SERS system presents a robust and efficient detection method, several considerations must be noted in order to improve its performance. First, the SERS effect is significantly quenched when targeting nanoplastics with the surface charge opposite to the AuNPs incorporated in the filter paper matrix. Thus, its future design can include the use of positively charged AuNPs as a second set of testing, for effective detection of negatively charged PSNPs. Similarly, the presence of NaCl quenched the SERS effect indicating future application may require a desalination step (*e.g.*, dialysis) prior to analyte addition onto filter paper and subsequent Raman measurements. Lastly, this study showed the correlation between the improved Raman enhancement and particle size distribution (or the optimum synthetic condition to achieve this). However, to further establish this structure-performance relationship and desirable synthetic conditions, more systematic studies are warranted with a wide range of AuNP size and size distribution.

It is also established that nano-particulates in complex environmental and biological fluids are covered with “corona” layer(s) of the pre-existing molecules.^1^ The capability of SERS systems (not limited to filter paper-based) to detect both nanoplastics and corona layer (*i.e.*, whether SERS hot-spot can penetrate through the corona layer) is yet to be investigated in depth. Given the importance of identifying the molecular compositions of the corona layer in the risk assessment framework,^1^ it is important that future studies will tackle this challenge. Additionally, the future studies can effectively use chemometrics to help eliminating the cellulose background signals and distinguish between different plastic types (and other molecules).

In summary, this study demonstrated the first proof of principle for using filter paper as a supporting matrix of SERS active NPs to detect 20 nm sized PSNPs. A reliable LOD was 10 μg mL^−1^ was achieved, in a rare case, we observed the successful detection of PSNPs at 5 μg mL^−1^. While strong enhancements (EF = ∼3050 with ∼50% variance) for PS(+)20 was confirmed with AuNP2 (batch of which that showed the most narrow size distribution), the extent of which was significantly reduced for larger nanoplastics (PS(+)200) or completely quenched for negatively charged PSNPs (PS(−)20 and PS(−)200). While uniformity of the SERS matrix is the key to the reproducible and effective enhancement, the present system demonstrates that the heterogenous AuNP/nanoplastic aggregate formation is the essential condition to achieve these. Nevertheless, this simple system offers great potential in detecting nanoplastics and may be a forerunning candidate as a standardised methodology for plastic nanotoxicology research.

## Author contributions

Shinji Kihara: conceptualisation, data curation, formal analysis, investigation, funding acquisition, project administration, writing – original draft, review & editing. Andrew Chan: conceptualisation, formal analysis, investigation, methodology, writing – original draft. Eugene In: methodology, investigation, visualisation, writing – original draft, Nargiss Taleb: methodology, investigation, visualisation, writing – original draft, Cherie Tollemache: methodology, writing – review & editing. Samuel Yick: conceptualisation, writing – review & editing. Duncan McGillivray: conceptualisation, funding acquisition, supervision, writing – review & editing.

## Conflicts of interest

There are no conflicts to declare.

## Supplementary Material

RA-012-D2RA03395J-s001
