# Supplementary material for: Detecting polystyrene nanoplastics using filter paper-based surface-enhanced Raman spectroscopy
Source: RSC Adv. 2022 Jul 15;12(32):20519–22. doi: 10.1039/d2ra03395j (PMC9286027; doi:10.1039/d2ra03395j)
Supplement: RA-012-D2RA03395J-s001 [file RA-012-D2RA03395J-s001.pdf]

Electronic Supplementary Material (ESI) for RSC Advances.
This journal is © The Royal Society of Chemistry 2022

Electronic Supplementary Information

Detecting nanoplastics with filter paper-based surface-enhanced Raman Spectroscopy

Shinji Kihara[a,b], Andrew Chan[a,b], Eugene In[a], Nargiss Taleb[a], Cherie Tollemache[a], Samuel Yick[a,b], and Duncan J. McGillivray[a,b*]

[a] School of Chemical Sciences, The University of Auckland, Auckland 1010, New Zealand

[b] The MacDiarmid Institute for Advanced Materials and Nanotechnology, Wellington 6140, New Zealand

Experimental Section:

Chemicals and Materials

Trisodium citrate dihydrate ($Na_3C_6H_5O_7·2H_2O$, 99.0%, ECP) and gold(III) chloride hydrate ($HAuCl_4·3H_2O$, 99.995%, Sigma-Aldrich) were used without further purification to synthesise AuNPs. In all experimental investigations, ultrapure water (resistance = 18.2 MΩ·cm) was the solvent.

A range of PS nanoparticles were used as model nanoplastics, with different sizes (~20 and 200 nm) and surface functionalisation (carboxylic acid and amidine-modified, PS(-) and PS(+), respectively). The following PS nanoparticles were purchased from Thermo Fischer Scientific: PS(-) (0.02 µm, 4% *w/v*), PS(-) (0.2 µm, 4% *w/v*), PS(+) (0.02 µm, 4% *w/v*), and PS(+) (0.2 µm, 4% *w/v*). Filter paper (Grade: MS 1, 110 mm diameter, lot No. 19-091-1) was sourced from MicroAnalytix (Auckland, New Zealand).

Preparation of Au nanoparticles (AuNPs)

AuNPs were prepared via the Reversed Turkevich method with modifications[1]. In brief, chloroauric acid solution (167 µL, 25 mM) was added to a boiling trisodium citrate solution (25 mL, 2.2 mM) in a two-neck round-bottom flask (100 mL) under reflux. The reaction was left to proceed for 15 min until the solution did not undergo any further colour change from pink-red that indicates the complete formation of AuNPs. The synthesised AuNP colloidal suspension was collected and stored in sealed Schott bottle at 4 °C. Various molar ratios of trisodium citrate and chloroauric acid solution were varied to achieve the best size and shape monodispersity of AuNPs. In particular, the variable was chloroauric acid solution volume (167-500 µL, 25 mM) added to the reaction vessel. A summary of reaction conditions is listed in Table 1.

Table S1. The AuNP series synthesised in this work varied the concentration of chloroauric acid in the reaction mixture (0.17-0.81 mM) with a fixed trisodium citrate concentration and volume (2.2 mM, 25 mL). The calculation of AuNP concentration (in nM) assumed 100% yield and were spherical in shape.

AuNP batch name	Chloroauric Acid Solution			
	Volume of chloroauric acid added / mL	Final chloroauric acid concentration / mM	Final AuNP concentration / nM	Final AuNP concentration / mg mL^{-1}
AuNP1	0.167	0.17	0.39	0.05
AuNP2	0.334	0.33	0.78	0.1
AuNP3	0.500	0.49	1.2	0.15
AuNP4	0.668	0.65	1.6	0.20
AuNP5	0.835	0.81	2.0	0.25

UV-Vis spectroscopy

A Shimadzu UV-2600 UV-Vis was used to record UV-Vis absorbance measurements conducted in quartz cuvettes (1.0 cm path length). Spectra were recorded in the wavelength range of 200-1400 nm, with a slit width of 5.0 nm and 0.2 nm spectral resolution.

Transmission Electron Microscopy (TEM)

Bright-field images were taken on a FEI Tecnai G2 F20 TWIN TEM with the field emission gun operated at an accelerating voltage of 200 kV and emission current of 102 µA. Prior to analyses, 1 µL of aqueous PS nanoplastics (40 ng mL^{-1}) or AuNPs were drop-casted onto glow discharge treated Cu-TEM grids (3 mm, 400-mesh, laced with carbon and formvar) and left to dry in ambient conditions for 15 min. For optimised image contrast, the microscope condenser aperture C2 = 100 µm (#3), objective aperture = 20 µm (#2), spot size = 5, and the objective lens strength ~88.1% to operate in the under-focussed regime. Particle size analyses from bright-field images were performed using the inbuilt 'Analyze Particles' macro of ImageJ[2].

Dynamic Light Scattering (DLS) and Zeta Potential Measurements

Particle size distribution and zeta potential analyses were conducted with a Malvern Instruments Nanozetasizer ZS equipped with a 633 nm laser. The size distribution of AuNPs and PS nanoplastics were measured in non-invasive back scatter (NIBS) mode with the photomultiplier detector placed at 173° relative to the incident beam. Hydrodynamic diameter and polydispersity index (PDI) were estimated with a cumulant fit. For zeta potential determination, the Smoluchowski approximation was used.

Preparation of filter paper-based SERS substrate and Raman Spectroscopy

Prior to deposition of analytes onto filter paper, PSNPs were premixed with AuNPs (0.39 mM) in equal parts. The colloidal mixture of AuNPs and PSNPs was then added dropwise to filter paper. For single application of analytes, a 50 µL sample volume was used and left to air dry for at least 2 h. For multiple applications, this process was repeated four times.

Raman spectra were collected using a Labram HR Evolution confocal Raman spectrometer (Horiba, Japan), coupled with a CCD camera. The samples were excited by an external-cavity diode laser (785 nm), operating at a power of approximately 10, 50, or 100 mW focussed through a × 50 microscope objective lens (NA = 0.5). Raman spectral positions were calibrated to the Si peak position of 520.7 cm^{-1} using × 100 microscope objective lens (NA = 0.9).

Spectra were collected from 200-1800 cm^{-1}, with ten accumulations for reference materials (e.g., polystyrene, trisodium citrate, and filter paper) using 30 s acquisition time with a confocal hole width fixed at 500 µm. To calculate the enhancement factor (EF) of the AuNPs, spectra were collected with ~50 mW laser power, 20 s acquisition time, and averaging three accumulations. For all other measurements, the laser power was fixed at ~10 mW, with 120 acquisition time, and two accumulations to average.

Baseline removal was performed using LabSpec 6 Software (Horiba Scientific, Japan), by a polynomial equation which best fits each spectrum (typically with 5 orders and 116 points). Furthermore, the cosmic ray contribution was eliminated by applying de-spiking function with a3-5 pixel threshold.

For calculating enhancement factors (EF) of different AuNP batches, reference measurement was conducted for PS(+)20 (200 mg mL^{-1}) deposited to a filter paper, which was then compared with the spectra collected for a mixture of AuNPs (0.5 mg mL^{-1}) and PS(+)20 (1.0 mg mL^{-1})

in a filter paper. The EF was then calculated by comparing the intensities of PS characteristic peak at 1004 cm^{-1}, for the reference (*I(reference)*) and enhanced (*I(enhanced)*) measurements, using the following formula:

$$EF = \frac{I(enhanced)}{I(reference)} \times \frac{C(enhanced)}{C(reference)}$$

where the *c(enhanced)* is 1.0 mg mL^{-1} and *c(reference)* is 200 mg mL^{-1}, denoting the corresponding PSNP concentration used. Median (from at least 10 independently sampled spots) of experimentally collected intensity was used in *I(enhanced)* for each AuNP batch (Figure 1c in the main text). Particular attention was paid to fix the key instrumental parameters (e.g., laser power = 50 mW and confocal hole = 500 μm) so the Raman intensity variation is due to the enhancement effect from the AuNP and sampled spots.

Results:

Table S2. A summary table for the physical properties (diameter, hydrodynamic diameter, polydispersity index (PDI), and zeta potential) of the synthesised AuNP batches and PSNPs. The diameters of the PSNPs measured by small-angle neutron scattering (SANS) were investigated in our previous work[3, 4].

	Diameter, TEM / nm	Aspect ratio	Diameter SANS / nm[3, 4]	Hydro-dynamic diameter, DLS / nm	PDI DLS	Zeta potential / mV
AuNP1	21.9 (±6.7)*	1.08 (±0.10)*	N/A	23	0.45	-36 (±18)
AuNP2	23.2 (±4.2)*	1.07 (±0.11)*	N/A	24	0.44	-35 (±21)
AuNP3	23.2 (±5.8)*	1.11 (±0.11)*	N/A	37	0.81	-36 (± 11)
AuNP4	27.2 (±4.3)*	1.10 (±0.11)*	N/A	22	0.50	-43 (± 23)
AuNP5	25.6 (±5.6)*	1.07 (±0.06)*	N/A	26	0.50	-30 (± 13)
PS(+)20	N/A	N/A	23 (±1.0)	40	0.12	+27 (± 8.5)
PS(+)200	N/A	N/A	222 (±1.0)	234	0.013	+40 (± 7.0)
PS(-)20	N/A	N/A	22 (±0.1)	34	0.15	-42 (± 11)
PS(-)200	N/A	N/A	217 (±0.1)	221	0.04	-56 (± 4.9)

*** The diameters of AuNPs were estimated by the centre position of the Gaussian distribution of measured diameter and the full-width half maxima (distributions shown in**

Fig. S3).

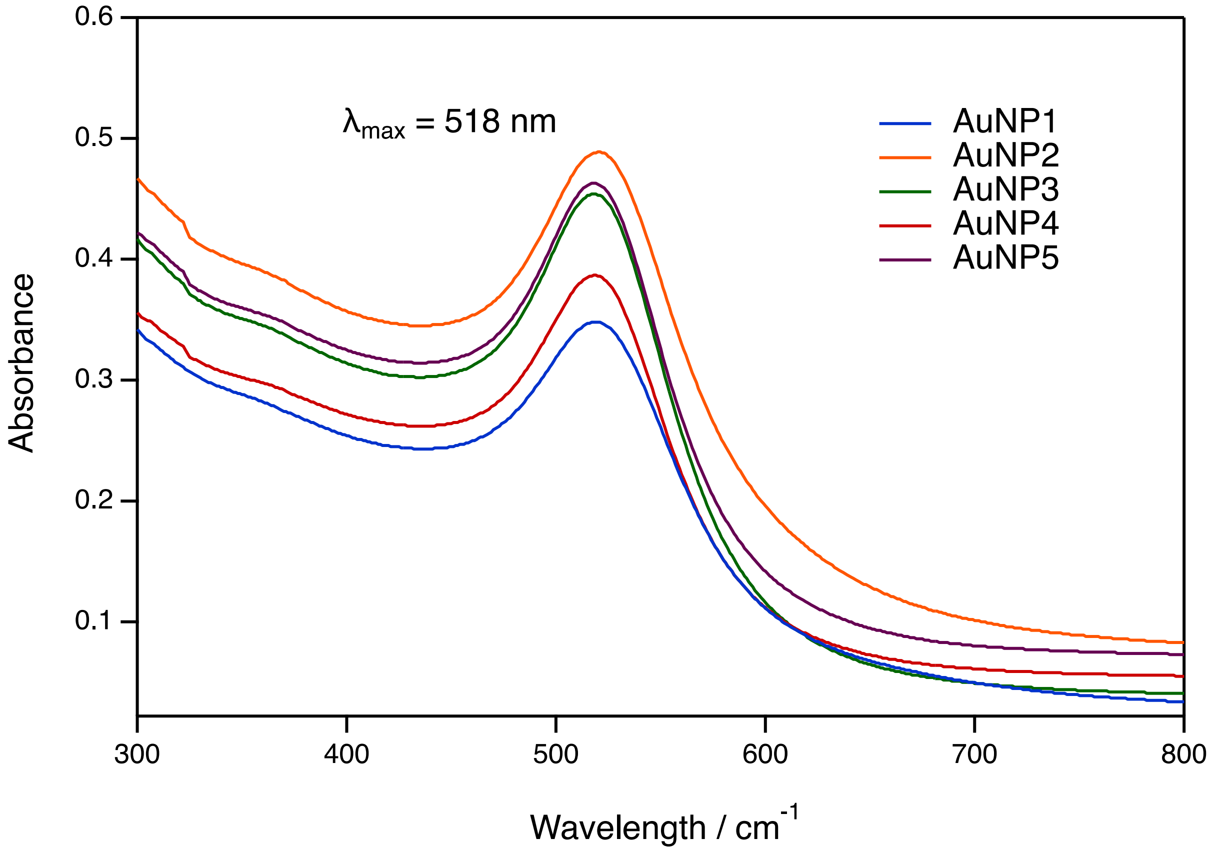

Fig. S1. UV-Vis spectra for AuNP batches with their concentrations corrected to AuNP1 (0.05 mg mL^{-1}).

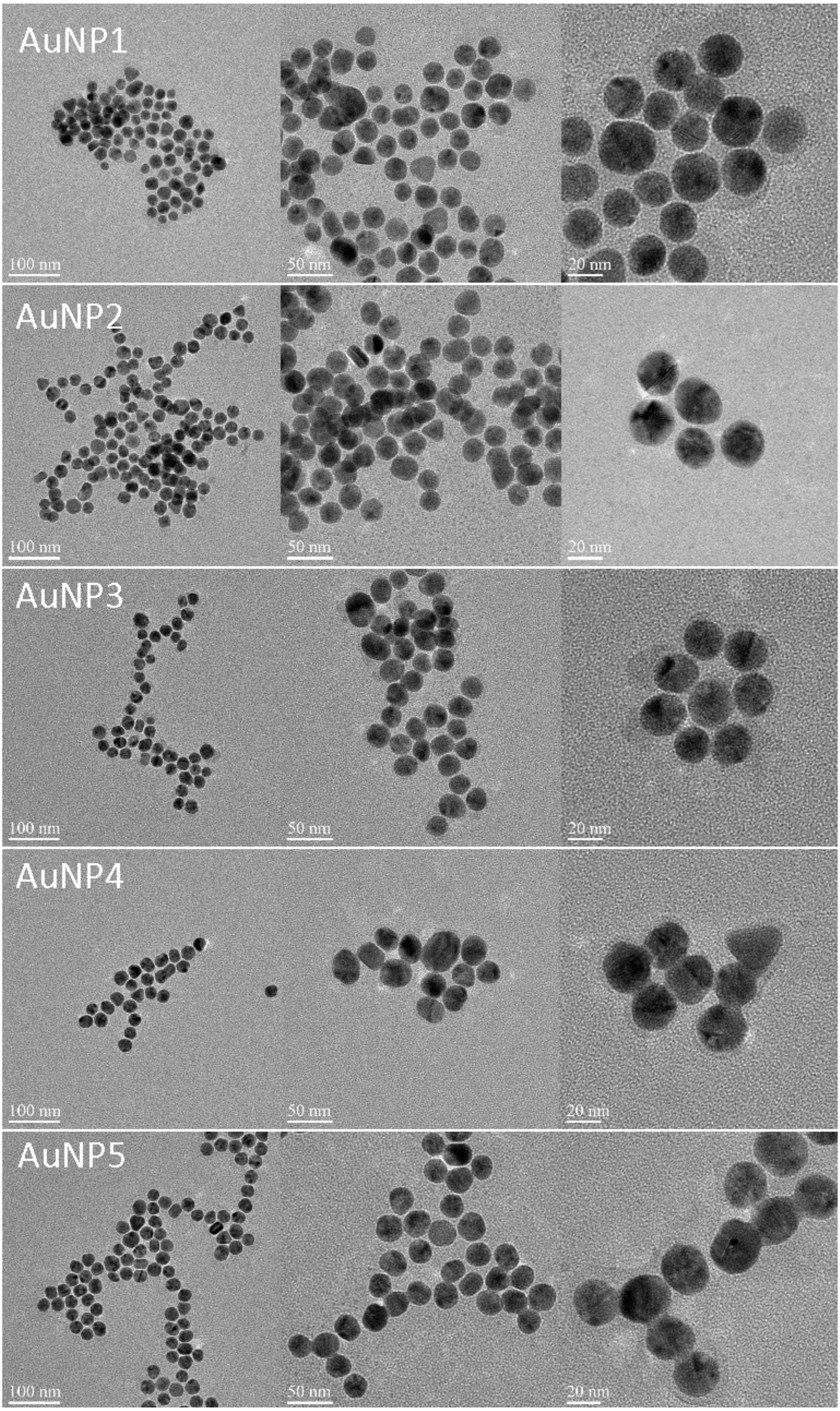

Fig. S2. Representative transmission electron micrographs of AuNP batches synthesised in this work.

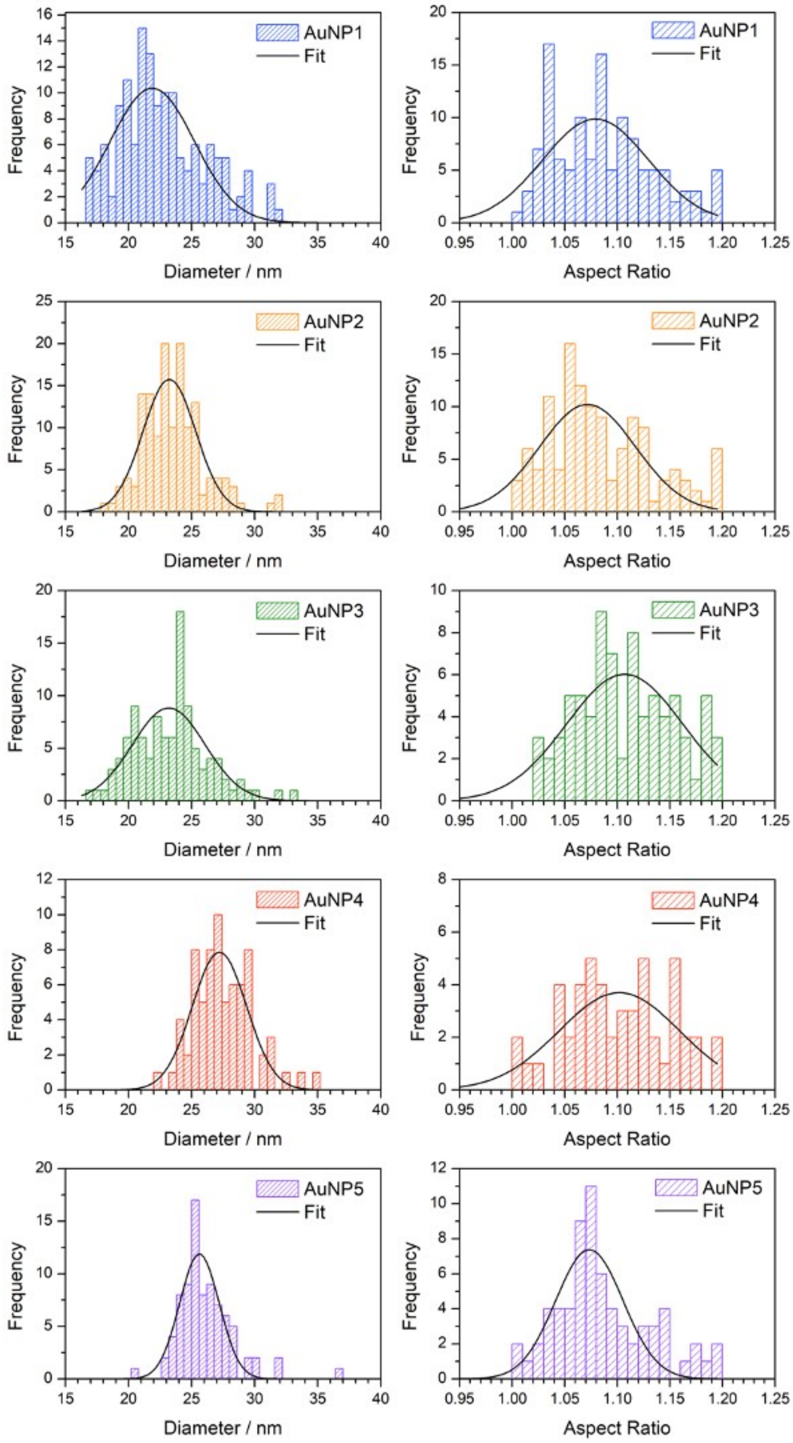

Fig. S3. Particle diameter and aspect ratio distributions of AuNP batches. The distributions were constructed from measurements of AuNPs imaged by TEM. Gaussian fits are shown as black curves on each histogram.

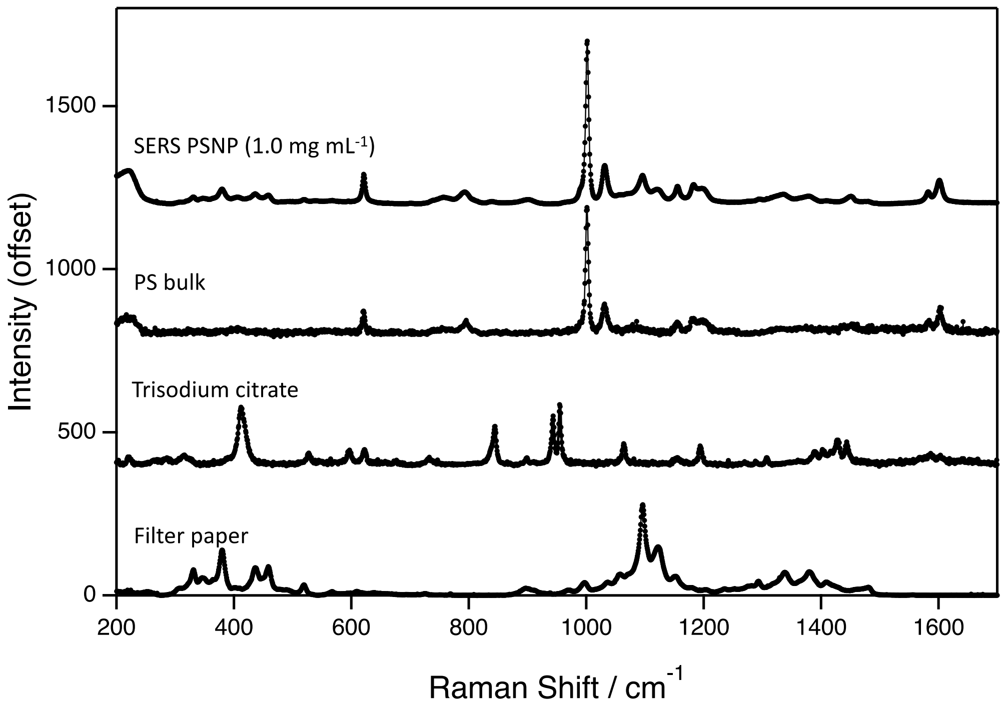

Fig. S4. Raman spectra for signal-enhanced PSNP (PS(+)20, 1.0 mg mL^{-1}) with AuNP2 on filter paper, PS bulk, trisodium citrate, and bare filter paper.

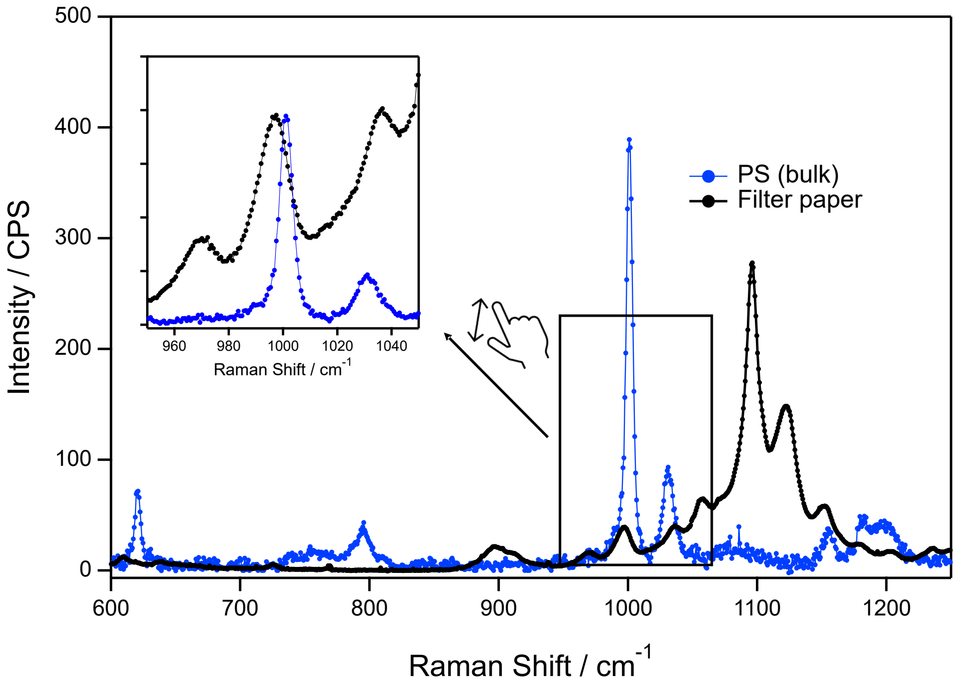

Fig. S5. Raman spectra for PS (bulk) and filter paper and (inset) the Raman peak intensities were normalised to the characteristic PS peak at 1004 cm^{-1} to highlight the peak position differences.

Table S3. The list of median CPS detected at 1004 cm^{-1} (characteristic PS peak), calculated EF, and inter-quartile range for each AuNP batch.

	AuNP1	AuNP2	AuNP3	AuNP4	AuNP5
Median CPS at 1004 cm^{-1}	108.5	304.5	123	100.5	176.5
EF	1085	3045	1230	1005	1765
Inter-quartile range	714	1440	875	810	833

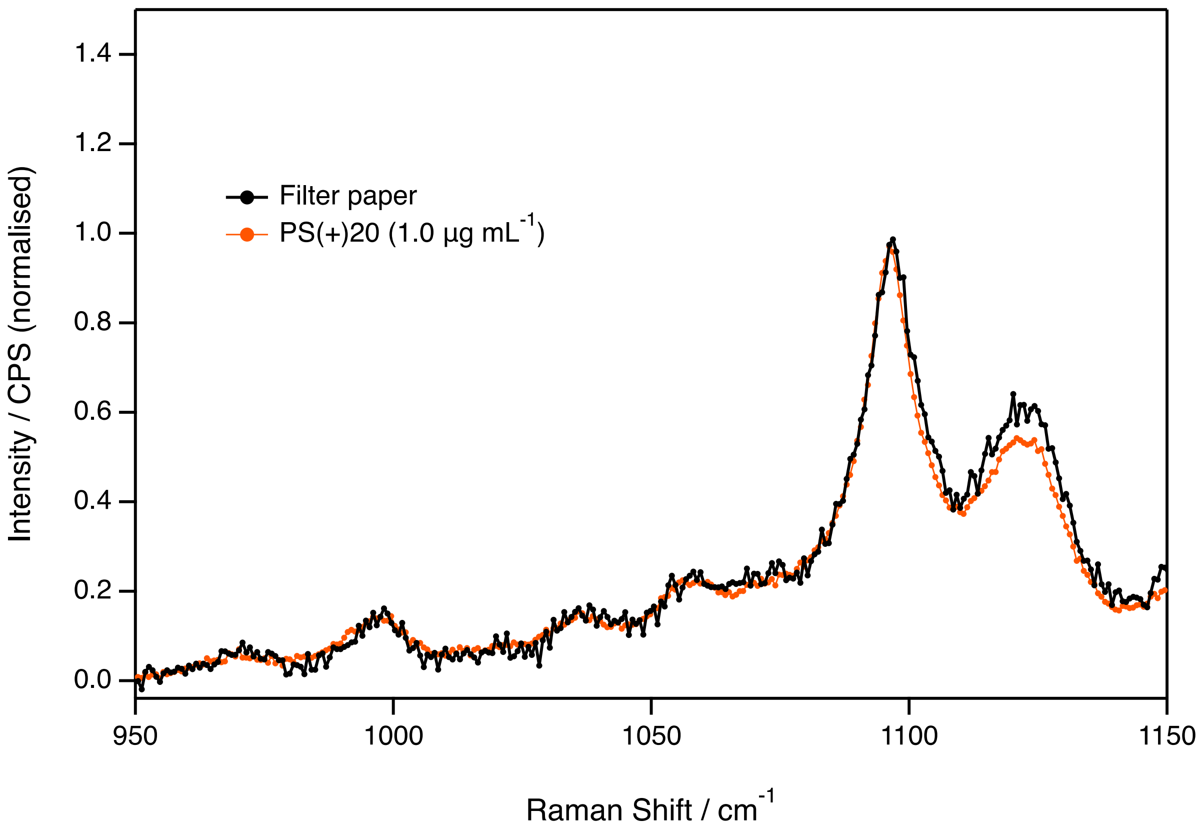

Fig. S6. Surface-enhanced Raman spectra for filter paper and PS(+)20 at 1.0 g mL^{-1} with AuNP2 drop casted on to filter paper, versus a bare filter paper. The Raman intensity was normalised to the peak at 1075 cm^{-1}.

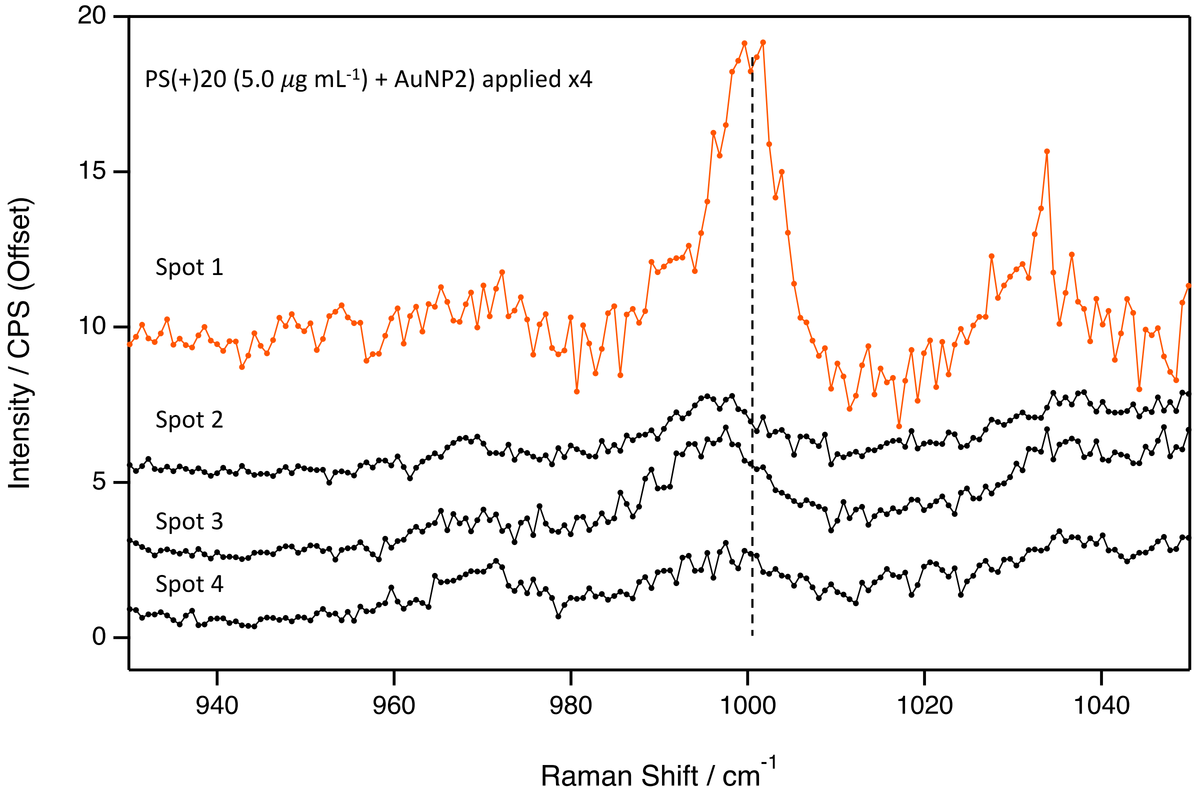

Fig. S7. Surface-enhanced Raman spectra for PS(+)20 (5.0 µg mL^{-1}) with AuNP2 drop casted onto filter paper (total of four aliquots of analytes were applied). Spot 1 showed successful enhancement of PS signals while other sampled spots did not (spot 2-4).

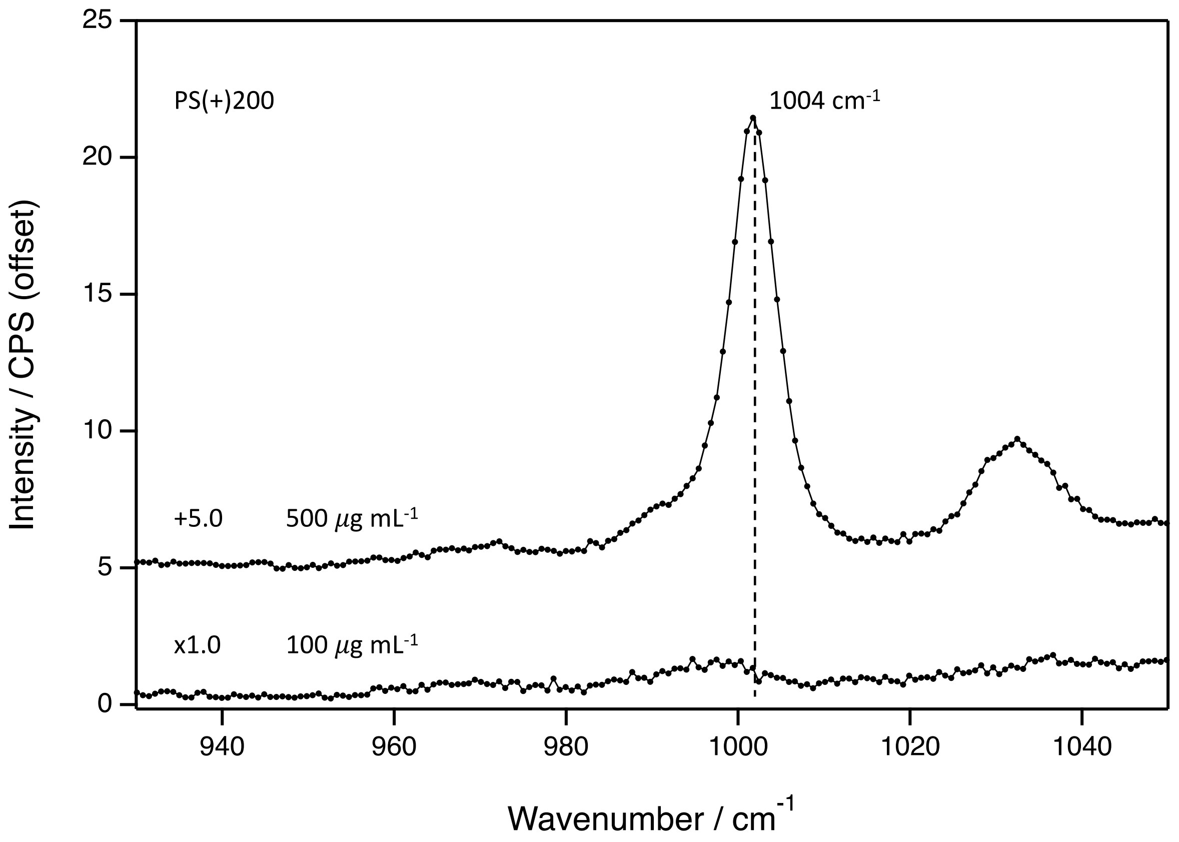

Fig. S8. Surface-enhanced Raman spectra for PS(+)200 at 500 and 100 µg mL^{-1} with AuNP2 drop-casted onto filter paper.

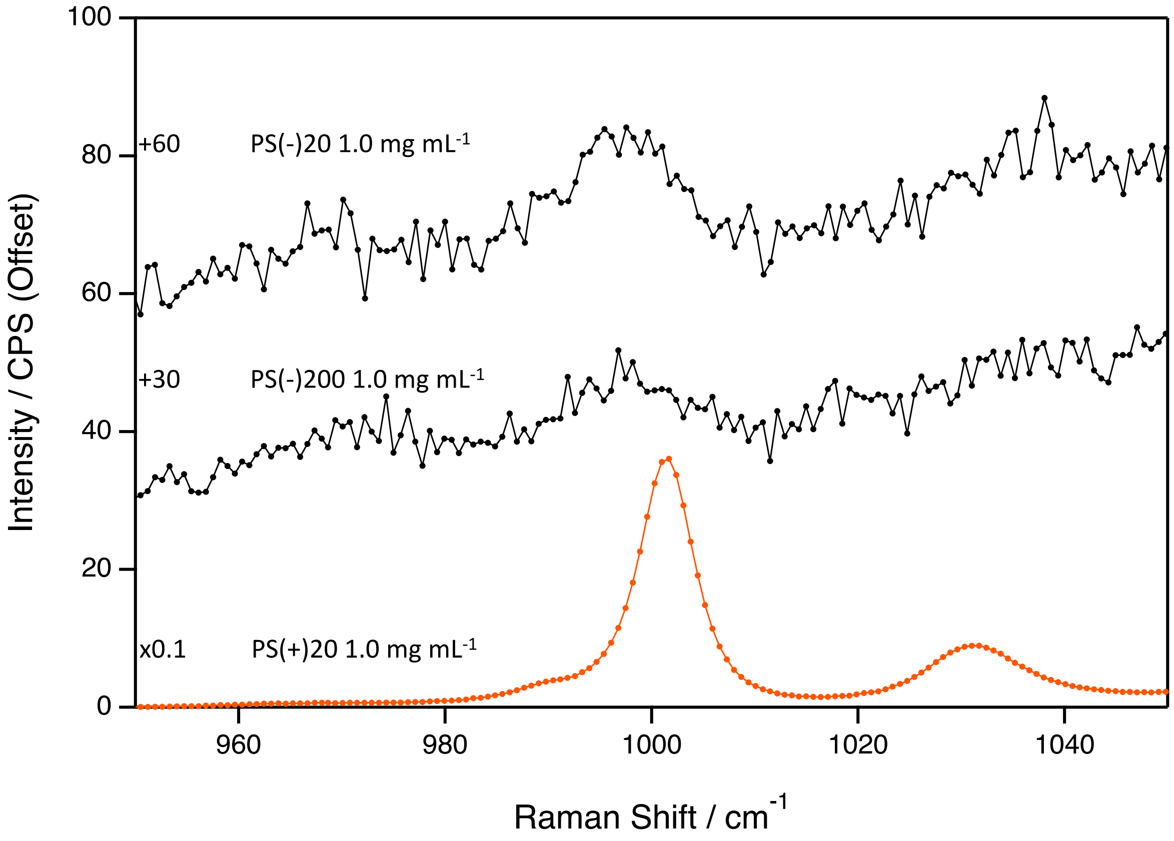

Fig. S9. Surface-enhanced Raman spectra for PS(-)20 and PS(200) at 1.0 mg mL^{-1} with AuNP2. A spectrum for PS(+)20 at 1.0 mg mL^{-1} (intensity multiplied by 0.1 for clarity) for reference.

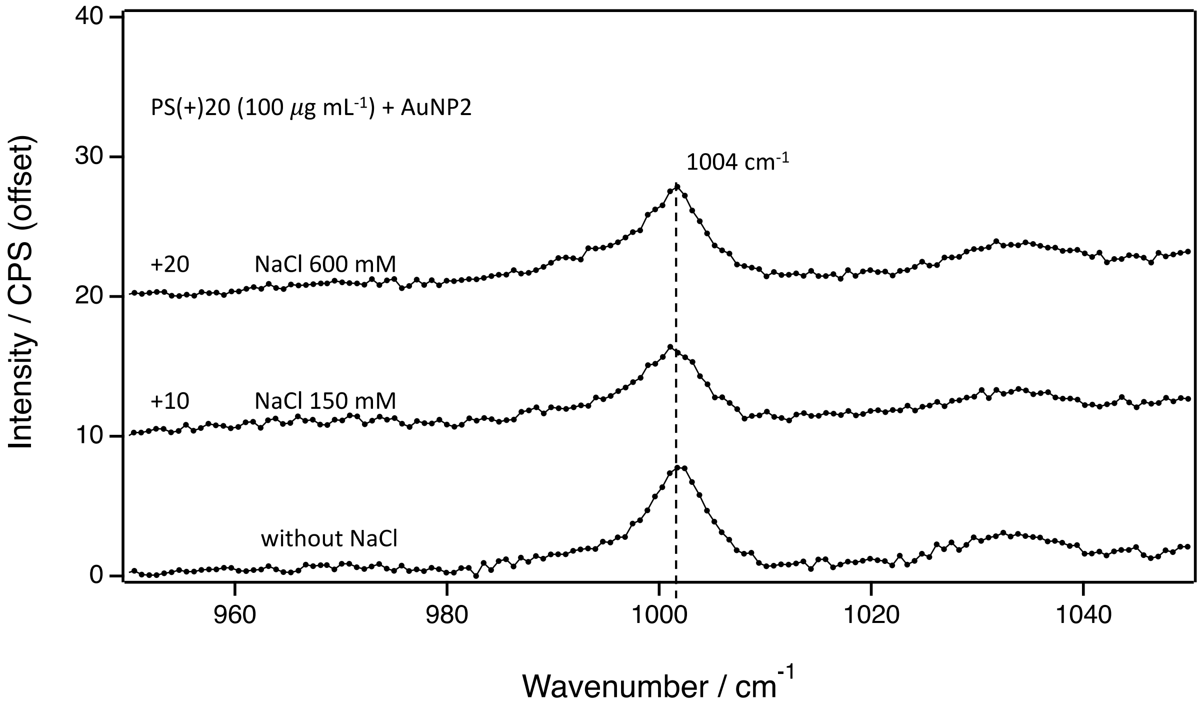

Fig. S10. Surface-enhanced Raman spectra for PS(+)20 with AuNP2 and NaCl (0.0, 150, or 600 mM) on filter paper.

References

1. Kimling, J.; Maier, M.; Okenve, B.; Kotaidis, V.; Ballot, H.; Plech, A., Turkevich method for gold nanoparticle synthesis revisited. *The Journal of Physical Chemistry B* **2006,** *110* (32), 15700-15707.
2. Schneider, C. A.; Rasband, W. S.; Eliceiri, K. W., NIH Image to ImageJ: 25 years of image analysis. *Nature methods* **2012,** *9* (7), 671-675.
3. Kihara, S.; van der Heijden, N. J.; Seal, C. K.; Mata, J. P.; Whitten, A. E.; Köper, I.; McGillivray, D. J., Soft and Hard Interactions between Polystyrene Nanoplastics and Human Serum Albumin Protein Corona. *Bioconjugate Chem.* **2019,** *30* (4), 1067-1076.
4. Kihara, S.; Ghosh, S.; McDougall, D. R.; Whitten, A. E.; Mata, J. P.; Köper, I.; McGillivray, D. J., Structure of soft and hard protein corona around polystyrene nanoplastics—Particle size and protein types. *Biointerphases* **2020,** *15* (5), 051002.
